# Supplementary material for: Exploration of the Binding Site of Arachidonic Acid in gp63 of Leishmania mexicana and in Orthologous Proteins in Clinically Important Parasites
Source: Pathogens. 2024 Aug 25;13(9):718. doi: 10.3390/pathogens13090718 (PMC11434690; doi:10.3390/pathogens13090718)
Supplement: Supplementary file 1 [file pathogens-13-00718-s001.zip › pathogens-3150796-supplementary.pdf]

Description	E value	Query cover %	Identity %	Accession
*Trypanosoma cruzi (*surface protease GP63 putative*)*	$1e^{-111}$	86	37.86	XP_817808.1
Entamoeba histolytica (cell surface protease GP63 putative*)*	$3e^{-37}$	71	27.43	XP_652632.1
Entamoeba dispar (hypothetical protein)	$3e^{-35}$	81	25.86	XP_001740726.1
Entamoeba invadens (hypothetical protein)	$1e^{-31}$	52	27.59	XP_004184102.1
Acanthamoeba castellanii (leishmanolysin, putative)	$5e^{-45}$	52	32.75	XP_004337275.1
Naegleria fowleri (hypothetical protein)	$2e^{-29}$	52	28.24	XP_044566011.1

Material Supplementary Table 1. Blast analysis, performed in the reverse of what was obtained in Table 1. In this result it is evident that the E values remain similar to those shown previously (Table 1). In this way, it is demonstrated that proteins from different organisms are reciprocal results and, therefore, orthologs.
