# Supplementary material for: Exploration of the Binding Site of Arachidonic Acid in gp63 of Leishmania mexicana and in Orthologous Proteins in Clinically Important Parasites
Source: Pathogens. 2024 Aug 25;13(9):718. doi: 10.3390/pathogens13090718 (PMC11434690; doi:10.3390/pathogens13090718)
Supplement: Supplementary file 1 [file pathogens-13-00718-s001.zip › pathogens-3150796-supplementary2.pdf]

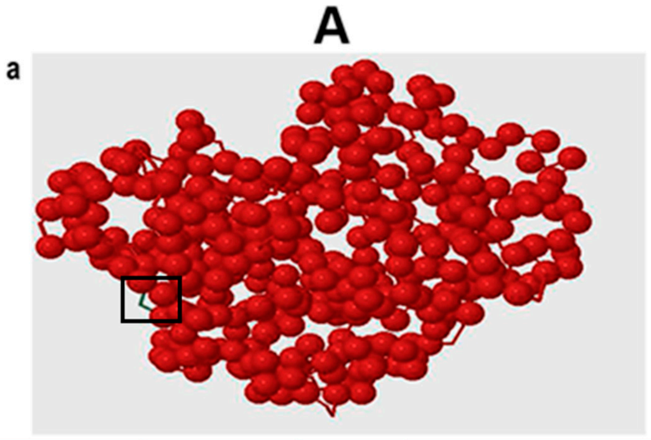

b

Description	Query Cover	E Value	Percentage Identity	Access number
Leishmania major (GP63, leishmanolysin)	97%	0	81.06%	XP_001681376.1

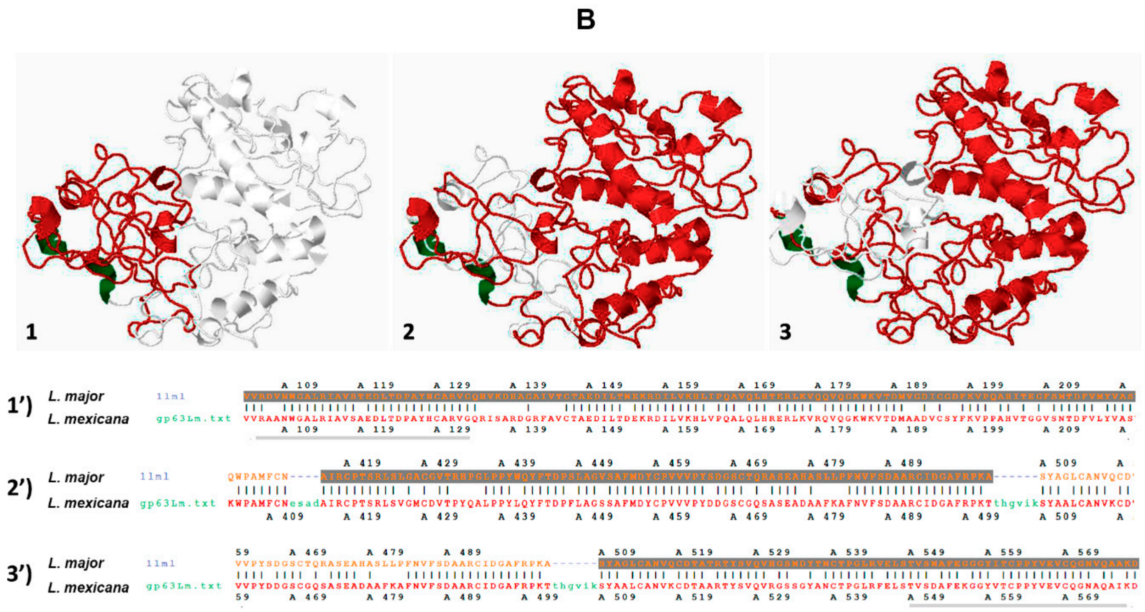

Supplementary materials Figure 1. Homology Model. The structural model of gp63 from *L. mexicana* was built by comparative modeling using the crystal structure of gp63 of *L. major* (PDB ID: 1LML) as the template. Subsequently, the structural similarity between both proteins was determined using the bioinformatics tool TopMach-web. In (Aa), the similarity of gp63 from *L. mexicana* and leishmanolysin from *L. major* is shown. The different sequences between both proteins are illustrated in green (indicated in a box) and the similar sequences are shown in red. In (Ab), the values obtained by the Blast analysis between both gp63 are shown. In (B), in the upper panel, the structural comparison (3D), by regions (1, 2, 3), of both proteins using the TopMach-web software is shown. The similar regions are in red and the different regions between both proteins are in green. At the bottom (1′, 2′, 3′) the alignment of the sequences that correspond to similar sequences is shown, marked in red, and the different sequences between both proteins are marked in green.
